# Predictors of Invasive Adenocarcinomas among Pure Ground-Glass Nodules Less Than 2 cm in Diameter

**DOI:** 10.3390/cancers13163945

**Published:** 2021-08-05

**Authors:** Wen-Chi Hsu, Pei-Ching Huang, Kuang-Tse Pan, Wen-Yu Chuang, Ching-Yang Wu, Ho-Fai Wong, Cheng-Ta Yang, Yung-Liang Wan

**Affiliations:** 1 Department of Medical Imaging and Intervention, Linkou Chang Gung Memorial Hospital, College of Medicine, Chang Gung University, Taoyuan 333, Taiwan; nkuj123321@gmail.com (W.-C.H.); spookie@cgmh.org.tw (P.-C.H.); pan0803@cgmh.org.tw (K.-T.P.); hfwong@adm.cgmh.org.tw (H.-F.W.); 2 Department of Anatomic Pathology, Linkou Chang Gung Memorial Hospital, College of Medicine, Chang Gung University, Taoyuan 333, Taiwan; s12126@cgmh.org.tw; 3 Division of Thoracic and Cardiovascular Surgery, Linkou Chang Gung Memorial Hospital, College of Medicine, Chang Gung University, Taoyuan 333, Taiwan; wu.chingyang@gmail.com; 4 Department of Thoracic Medicine, Chang Gung Memorial Hospital, Taoyuan 333, Taiwan; yang1946@cgmh.org.tw

**Keywords:** ground-glass nodule, CT, predictor, invasive adenocarcinoma, non-invasive adenocarcinoma, pre-invasive lesion

## Abstract

**Simple Summary:**

Benign lesions, atypical adenomatous hyperplasia, and malignancies such as adenocarcinoma in situ, minimally invasive adenocarcinoma, and invasive adenocarcinoma may feature pure ground-glass nodules on chest CT images, and the prognosis of patients with invasive adenocarcinoma is worse than others. The early detection and adequate management of invasive adenocarcinoma is crucial, but the pathology diagnosis of small nodules is difficult to obtain without surgery. Our study aimed to analyze the CT characteristics of pure ground-glass nodules <2 cm for the identification of invasive adenocarcinomas. A total of 181 nodules in 171 patients were enrolled. The larger size, lobulation, and air cavity were significantly more common in invasive adenocarcinoma. The air cavity is the significant predictor in multivariate analysis. In conclusion, the possibility of invasive adenocarcinoma is higher in a pure ground-glass nodules when it is associated with a larger size, lobulation, and air cavity.

**Abstract:**

Benign lesions, atypical adenomatous hyperplasia (AAH), and malignancies such as adenocarcinoma in situ (AIS), minimally invasive adenocarcinoma (MIA), and invasive adenocarcinoma (IA) may feature a pure ground-glass nodule (pGGN) on a thin-slide computed tomography (CT) image. According to the World Health Organization (WHO) classification for lung cancer, the prognosis of patients with IA is worse than those with AIS and MIA. It is relatively risky to perform a core needle biopsy of a pGGN less than 2 cm to obtain a reliable pathological diagnosis. The early and adequate management of patients with IA may provide a favorable prognosis. This study aimed to disclose suggestive signs of CT to accurately predict IA among the pGGNs. A total of 181 pGGNs of less than 2 cm, in 171 patients who had preoperative CT-guided localization for surgical excision of a lung nodule between December 2013 and August 2019, were enrolled. All had CT images of 0.625 mm slice thickness during CT-guided intervention to confirm that the nodules were purely ground glass. The clinical data, CT images, and pathological reports of those 171 patients were reviewed. The CT findings of pGGNs including the location, the maximal diameter in the long axis (size-L), the maximal short axis diameter perpendicular to the size-L (size-S), and the mean value of long and short axis diameters (size-M), internal content, shape, interface, margin, lobulation, spiculation, air cavity, vessel relationship, and pleural retraction were recorded and analyzed. The final pathological diagnoses of the 181 pGGNs comprised 29 benign nodules, 14 AAHs, 25 AISs, 55 MIAs, and 58 IAs. Statistical analysis showed that there were significant differences among the aforementioned five groups with respect to size-L, size-S, and size-M (*p* = 0.029, 0.043, 0.025, respectively). In the univariate analysis, there were significant differences between the invasive adenocarcinomas and the non-invasive adenocarcinomas with respect to the size-L, size-S, size-M, lobulation, and air cavity (*p* = 0.009, 0.016, 0.008, 0.031, 0.004, respectively) between the invasive adenocarcinomas and the non-invasive adenocarcinomas. The receiver operating characteristic (ROC) curve of size for discriminating invasive adenocarcinoma also revealed similar area under curve (AUC) values among size-L (0.620), size-S (0.614), and size-M (0.623). The cut-off value of 7 mm in size-M had a sensitivity of 50.0% and a specificity of 76.4% for detecting IAs. In the multivariate analysis, the presence of air cavity was a significant predictor of IA (*p* = 0.042). In conclusion, the possibility of IA is higher in a pGGN when it is associated with a larger size, lobulation, and air cavity. The air cavity is the significant predictor of IA.

## 1. Introduction

Lung cancer was the leading cause of cancer death worldwide in 2020, as well as the 1st and 3rd most common cancer in men and women, respectively. There were 2.2 million newly diagnosed cases and 1.7 million cancer deaths reported for lung cancer [1]. The National Lung Screening Trial (NLST) reported a reduction in lung cancer mortality with chest computed tomography (CT) screening in the participants with high cancer risk [2]. Since 2013, the Preventive Services Task Force (USPSTF) has recommended low-dose computed tomography for lung cancer screening [3]. With the increased application of chest CT for lung cancer screening, pure ground-glass nodules (pGGNs) were frequently found [4].

The cause of pGGN varied, including not only benign lesions such as focal interstitial fibrosis, infection, and inflammation, but also lung cancer and its precursors [5]. In 2011, the International Association for the Study of Lung Cancer (IASLC), American Thoracic Society (ATS), and European Respiratory Society (ERS) established a new categorization for adenocarcinoma of the lung, including preinvasive lesions with atypical adenomatous hyperplasia (AAH) and adenocarcinoma in situ (AIS), minimally invasive adenocarcinoma (MIA), and invasive adenocarcinoma (IA) [6]. AAH, AIS, MIA, and IA may feature pGGN [7,8]. With adequate surgical resection, the patients with AIS and MIA have 100% or near 100% disease-free survival [9,10,11], and those with localized IA were associated with a 5-year survival rate of 70–90% [12,13]. If left untreated, the median survival of early lung cancer is merely 13 months in patients with T1 disease [14]. Therefore, it is crucial to identify IA earlier for a better outcome.

It is a technical challenge to perform a core needle biopsy for a pGGN < 2 cm [15,16]. Therefore, some investigators have tried to identify IAs or malignant nodules presenting as pGGNs. Some authors attempted to differentiate preinvasive adenocarcinomas from IAs that present as pGGNs [4,17,18,19,20,21,22,23,24], while others investigated the correlation between the pathological findings and nodules presenting as solid, part-solid and pGGNs [25,26]. Our study aimed to analyze the CT findings of pGGN < 2 cm and differentiate IA from non-IA (NIA) for the appropriate management of pGGNs.

## 2. Materials and Methods

### 2.1. Patient Selection

**Figure 1:**
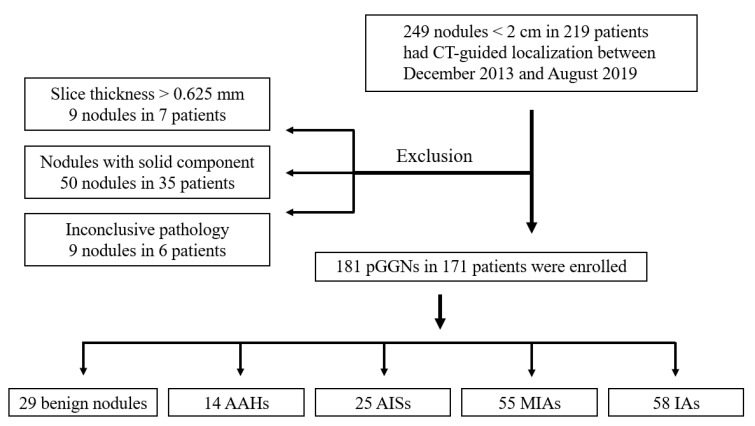
Flowchart of patient selection. CT-guided: computed tomography-guided; pGGNs: pure ground-glass nodules; AAH: atypical adenomatous hyperplasia; AIS: adenocarcinoma in situ; MIA: minimally invasive adenocarcinoma; IA: invasive adenocarcinoma.

This study was approved by the Institutional Review Board of Chang Gung Medical Foundation (IRB No: 202001934B0 issued on 9 November 2020) and in compliance with the Health Insurance Portability and Accountability Act. In a period of 69 months, from December 2013 to August 2019, patients who underwent preoperative CT-guided localization for lung nodule excision were retrospectively reviewed. The inclusion criteria were shown as follows: (1) pGGN with a mean diameter <20 mm. The mean diameter was defined as the average of the long axis and short axis diameters, measured on a lung window CT image. (2) Slice thickness of 0.625 mm on non-enhanced chest CT. (3) The presence of definite pathological diagnoses. According to the inclusion and exclusion criteria shown in the flowchart in Figure 1, the CT images with a slice thickness greater than 0.625 mm and nodules with solid components or inconclusive pathological results were all excluded. Both benign and malignant pGGNs were included in this study. Finally, a total of 181 nodules in 171 patients were enrolled in this study. The clinical data of each patient were recorded; these included age, gender, smoking history, drinking history, cancer history, and the pathological results of the nodules.

### 2.2. CT Examination

Patients were referred for preoperative localization by the surgeon. All CT-guided localization was performed using a helical CT scanner (BrightSpeed scanner GE Medical Systems, Milwaukee, WI, USA). The scanning parameters of the non-enhanced chest CT are as follows: (1) 120 kVp, (2) 100–250 mA, (3) beam pitch, 0.875–1.675, and (4) reconstructed slice thickness of axial images, including 0.625 mm and 5 mm. The image with 0.625 mm slice thickness was used for nodule analysis.

### 2.3. Pure Ground-Glass Nodule Analysis

**Figure 2:**
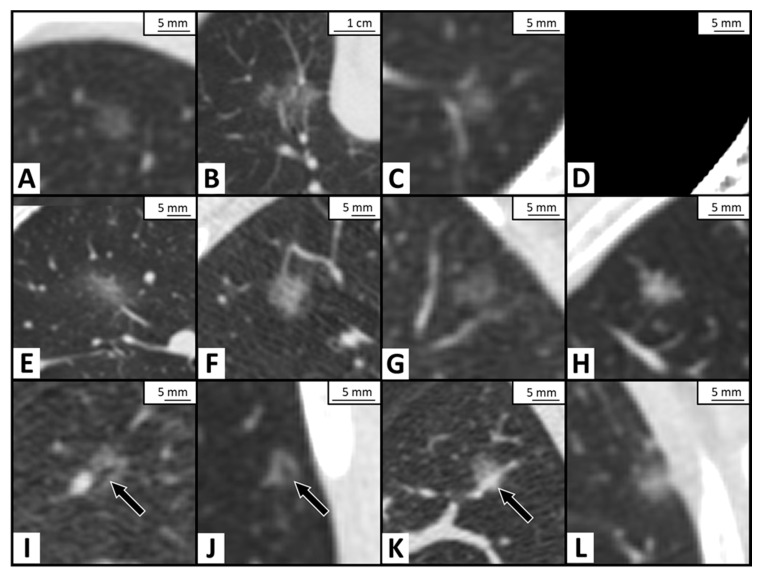
The illustration of imaging characteristic of the pure ground-glass nodule (pGGN) on computed tomography images. (**A**) Atypical adenomatous hyperplasia (AAH) with homogenous content, oval shape, well-defined interface, and smooth margin. (**B**) Minimally invasive adenocarcinoma (MIA) with irregular shape and vessel passing through. (**C**,**D**) Invasive adenocarcinoma (IA) with heterogeneous content, showing suspicious solid component in the center of nodule in lung window (window width/window level = 1500 HU/−600 HU) in C but invisible in mediastinal window (window width/window level = 350 HU/50 HU) in D. (**E**) Necrotizing granulomatous inflammation with the ill-defined interface. (**F**) IA with coarse margin and polygonal in shape. (**G**) IA with lobulation. (**H**) MIA with spiculation. (**I**) IA adjacent to a vessel with air-bronchogram (black arrow) in the nodule. (**J**) IA with vacuole sign (black arrow). (**K**) IA with type II vessel relationship, dilated vessel (black arrow) within the nodule. (**L**) Adenocarcinoma in situ with pleural retraction.

Two radiologists, who subspecialize in thoracic imaging (with 7 years and 37 years of experience, respectively), and were blinded to the pathology results and clinical information, reviewed all the nodules on the axial images with 0.625 mm slice thickness, obtained during the CT-guided localization. The divergence in image interpretation was resolved by consensus. Both the mediastinal window (window width/window level = 350 HU/50 HU) and lung window (window width/window level = 1500 HU/−600 HU) were used for interpretation. Pure GGN was defined as a nodule with hazy opacity, which did not obscure the underlying bronchial structures or pulmonary vessels under the lung window of non-enhanced CT [27]. The CT findings of the lung nodules were recorded and measured as follows: location (upper lobe versus not upper lobe), size-L (the maximal diameter in the long axis), size-S (the maximal short-axis diameter perpendicular to the long axis of size-L), size-M (the mean of long- and short-axis diameter), content (homogenous or heterogeneous), shape (oval, polygonal or irregular), interface (well-defined or ill-defined), margin (smooth or coarse), lobulation, spiculation, air cavity, vessel relationship, and pleural retraction. Of the content, pGGNs were further categorized into homogenous or heterogeneous: the homogenous nodules had homogeneous opacities in the lung window, and the heterogeneous nodules had suspicious solid components, which were only identified in the lung window but not in the mediastinal window [28]. Of the air cavity, both the vacuole sign and air–bronchogram were included, and the vacuole sign indicated tiny points, with translucent and air-density shadows in the nodules. In terms of the vessel relationship, type I was defined as vessels passing by or passing through the nodule, and type II was defined as distorted, dilated or engorged vessels within the nodule [29]. The imaging findings of the nodule are shown in Figure 2.

### 2.4. Pathological Diagnosis

All enrolled GGNs had a definite pathological diagnosis. Pathology results were classified into five groups: AAH, AIS, MIA, IA, and benign. The AAH, AIS, MIA, and IA were based on the International Association for the Study of Lung Cancer (IASLC)/American Thoracic Society (ATS)/European Respiratory Society (ERS) Classification of Lung Adenocarcinoma in Resection Specimens [6]. The benign group included the benign pathology result, except for AAH. Due to the worse prognosis of patients with IA, the benign lesions AAH, AIS, and MIA were designated as the NIA group.

### 2.5. Statistics

Statistical analyses were performed using the SPSS software package (Version 20.0. Armonk, NY, USA: IBM Corp.). Quantitative variables were expressed as mean ± standard deviation. Categorical variables were expressed as numbers and percentages. A comparison was performed among the five pathology groups: Benign, AAH, AIS, MIA, and IA. The quantitative variables, including age and nodule size, were compared with the Kruskal–Wallis test. Differences in the categorical variables, the patients’ gender, smoking history, drinking history, cancer history, nodule location, content, shape, interface, margin, lobulation, spiculation, air cavity, vessel relationship, and pleural retraction were compared with the chi-square test or Fisher test. A comparison between IA and NIA groups was performed with the Mann–Whitney U test for quantitative variables, and the chi-square or Fisher test was used for categorical variables. The receiver operating characteristic (ROC) curve of nodule size was created to determine the cut-off value used to predict IA, and the area under curve (AUC) value was calculated. The Youden index was performed to decide the optimal cut-off point. In the multivariate analysis, the imaging features with a *p*-value < 0.05 were included in the logistic regression with a forward stepwise selection, which was used to determine the predictors for IA. A *p*-value < 0.05 was considered statistically significant.

## 3. Results

### 3.1. Demographic Data and Nodule Information

The age of 171 patients (129 females) ranged from 25 to 81 years old (mean age 55.0 years old). There were 14 AAHs, 25 AIS’s, 55 MIAs, and 58 IAs, and 29 nodules were benign, comprising fibrosis, chronic inflammation, localized organizing pneumonia, and necrotizing granulomatous inflammation (Table 1). Ninety-eight (54.1%) nodules were located in the upper lobes. There was no significant difference among the five groups with respect to the demographic data, history of smoking, drinking and cancer, and the nodule location.

### 3.2. Imaging Characteristics Analysis

**Figure 3:**
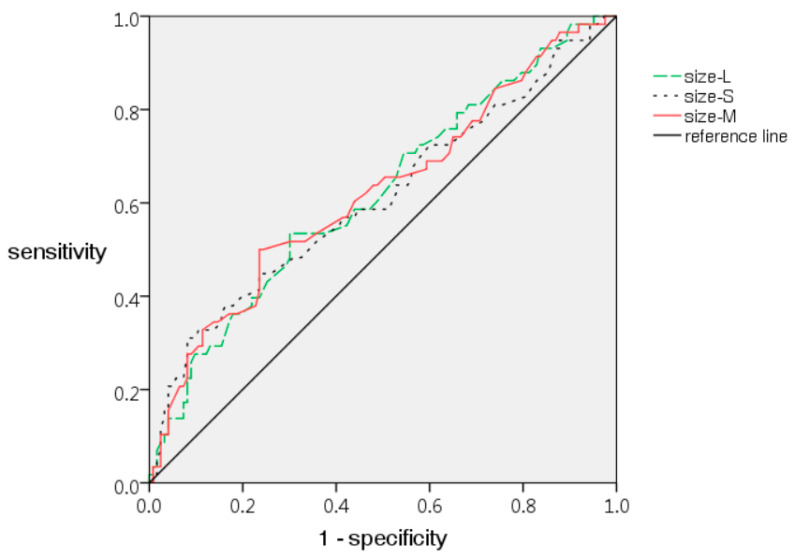
The receiver operating characteristic (ROC) curves of size-L, size-S, and size-M in discriminating invasive adenocarcinoma from other pathological results, with the area under curve (AUC) values of 0.620, 0.614, and 0.623, respectively. Size-L: the maximal diameter in the long axis; size-S: the maximal short-axis diameter perpendicular to the long axis of nodule; size-M: the mean value of long and short axis diameters.

Table 2 shows the CT imaging findings of the five pathology groups. Among the five groups, there were significant differences with respect to the size-L, size-S, and size-M (*p* = 0.029, 0.043, and 0.025, respectively). The sign of spiculation was only found in three (5.5%) of the 55 MIAs and one (1.7%) of 58 IAs without statistical significance. No significant difference was found among the five groups with respect to the other imaging findings. However, there were significant differences between the IA and NIA groups with respect to the size-L, size-S, and size-M (8.2 ± 3.4 mm vs. 6.8 ± 2.7 mm in size-L, 6.2 ± 2.6 mm vs. 5.2 ± 2.1 mm in size-S, and 7.2 ± 2.9 mm vs 6.0 ± 2.3 mm in size-M) with *p*-values of 0.009, 0.016, and 0.008, respectively (Table 3). The receiver operating characteristic (ROC) curve of each of the size-L, size-S, and size-M showed an AUC value of 0.620, 0.614, and 0.623, respectively (Figure 3). The cut-off value of 7.0 mm for size-M showed a sensitivity of 50.0% and a specificity of 76.4% in predicting IA. In terms of morphology, lobulation was significantly more prevalent in IA (6, 10.3%) than NIA (3, 2.4%), with a *p*-value of 0.031 (Table 3). The air cavity within nodules was also more frequently observed in IA (24, 41.4%) than in NIA (26, 21.1%), with a *p*-value of 0.004 (Table 3). In multivariate analysis with stepwise logistic regression, there was only one significant predictor for air cavity (odds ratio: 2.126; 95% confidence interval: 1.027–4.403, *p* = 0.042). The borderline *p*-value was shown in size-M (odds ratio: 1.146; 95% confidence interval: 0.999–1.315, *p* = 0.051) and lobulation (odds ratio: 4.382; 95% confidence interval: 0.981–19.583, *p* = 0.053).

## 4. Discussion

It may be relatively difficult to obtain a definite pathological diagnosis of pGGN less than 20 mm due to the technical challenges and potential risks of complications for pGGN when performing core needle biopsy [15,16]. The imaging features may play a crucial role in differentiating benign and malignant nodules. In Lung-RADS 1.1, recommended by the American College of Radiology (ACR), a pGGN of less than 30 mm is designated as negative. However, Kastner et al. reported that the increase in the allowable nodule size for pGGNs in category 2 from 20 mm (version 1.0) to 30 mm (version 1.1) showed no benefit, and one of the three down-categorized GGNs (version 1.1) was proved to be malignant (false-negative finding) [30]. In addition, the pGGNs classified into categories 2 or 3, based on Lung-RADS 1.1, were reported to have a higher malignancy rate than expected [31]. Therefore, it is worth investigating the imaging features to differentiate IA and NIA featuring a pGGN of less than 20 mm in diameter.

Benign GGNs were not included in studies that attempted to differentiate IA from pre-invasive lesions [4,17,18,19,20,21,22,23,24]. Some reports analyzed the differentiation of benign and malignant nodules that were solid, part-solid and GGN [25,26]. Our study aimed to investigate the potential predictors for differentiating IA from NIA, which featured a pGGN of less than 20 mm in diameter. In our study, there were significant differences among the five pathology groups (benign, AAH, AIS, MIA, and IA) with respect to size-L, size-S, and size-M. In comparison with NIA, IA had a significantly larger size-L, size-S, and size-M. The lobulation and air cavity were significantly more prevalent in IA than in NIA. Our multivariate analysis showed that the air cavity was a significant predictor of IA.

The correlation between nodule size and malignancy was frequently investigated. In the NELSON study, the probability of lung cancer was 0.4% for nodules smaller than 5 mm in diameter, and the malignancy rate was raised to 16.9% for nodules larger than 10 mm [32]. In the study based on the database of NSLT, the malignancy rate for GGNs with a size ranging from 10 to 19 mm was 6%, which is higher than 1.3% for GGN < 10 mm [31]. Qi et al. reported that the cut-off value of 11.5 mm for pGGNs ≤ 30 mm had a sensitivity of 75% and a specificity of 91.8% in identifying invasive adenocarcinoma among pGGNs [33]. Jin et al. reported that the optimal cut-off value of 10.5 mm had a sensitivity of 86.30% and a specificity of 61.90% in the differentiation of invasive and pre-invasive lesions [23].

In our study, there were significant differences in diameter among the five pathology groups and between the NIA and IA. The ROC curves of the size-L, size-S, and size-M were created with similar AUC values but associated with the highest figure of 0.623 for size-M in comparison with the 0.620 for size-L and 0.614 for size-S. The cut-off value of 7 mm in mean diameter showed a sensitivity of 50.0% and a specificity of 76.4% in differentiating NIA and IA. The difference in cut-off points and diagnostic accuracy in different series could be attributed to differences in the methods and materials, and further research in a larger sample size may be needed. The findings in previous reports and our results suggested the significance of size in the management of GGNs. The mean diameter of the long axis and short axis was recommended for the assessment of small nodules (<10 mm) by the Fleischner Society [34], and for the measurement of all nodules according to the Lung-RADS 1.1. Therefore, the measurement of both a long- and short-axis diameter is crucial for nodule evaluation.

Several studies discussed the CT morphology of the lung nodules, including the lobulation, spiculation, air-bronchogram, and vacuole sign [35,36,37,38]. Furuya et al. reported that 82% of the lobulated nodules and 97% of the spiculated nodules were malignant [39]. The lobulation and spiculation of pGGN were also more common in invasive lesions (IA and MIA) than in preinvasive lesions [4]. The lobulation is associated with more EGFR mutation in adenocarcinoma than the wild-type EGFR [40]. In our research, lobulation was more frequently found in IA (10.3%) than NIA (2.4%), with statistical significance (*p* = 0.031). However, there was no significant difference among the five pathology groups and between the IA and NIA with respect to spiculation. Nevertheless, the spiculation was only observed in MIA (3, 5.5%) and IA (1, 1.7%), and none was observed in the benign lesions, AAH, and AIS. The non-significance of spiculation in our study could probably be attributed to the limited number of spiculated nodules.

The air cavity, including air-bronchogram or vacuole, as seen in our study, was another CT characteristic related to malignancy. The air cavities are more commonly found in malignant rather than benign GGNs, and air-bronchograms are more frequently demonstrated in IA than in AIS [41,42]. In our study, the prevalence of the air cavity was higher in IA (24, 41.4%) than in NIA (26, 21.1%), with a *p*-value of 0.004. In our multivariate analysis, air cavity, size-M, and lobulation are significant or borderline significant predictors for IA, with a *p*-value of 0.042, 0.051, and 0.053, respectively. In our study, the air cavity in the nodules was found in 41.4% of IAs, and a total of 78.9% of NIA did not feature an air cavity; these findings are similar to those of another study, with a sensitivity of 53.7% and a specificity of 86.7% in differentiating malignancy from benignity among a total of 112 GGOs [41]. For usefulness in daily clinical practice, further investigation with a larger sample size and a better diagnostic performance may be required.

In terms of nodule content, Jin et al. classified the uniformity of ground-glass nodules into three categories, namely homogenous, less homogenous, and heterogeneous [23]. There were significant differences in the uniformity between preinvasive lesions and MIAs or invasive adenocarcinomas (*p* = 0.01, *p* < 0.05). Their study included 94 pGGNs, comprising 21 preinvasive lesions, 35 MIAs, and 38 IAs, but excluded benign nodules, except for AAHs. Chu et al. reported that invasive pGGNs (MIAs and IAs) were significantly more likely to be heterogeneous in density than preinvasive lesions (*p* < 0.05). Their study enrolled 172 pGGNs, including 14 AAHs, 59 AISs, 68 MIAs, and 31 IAs [4]. In our study, the prevalence of heterogeneous content was associated with a positive correlation with the nodule invasiveness, shown as follows: in AAH (28.6%), AIS (36.0%), MIA (45.5%), and IA (48.3%). However, heterogeneous attenuation was found in 41.4% of our benign pGGNs. The inclusion of all benign pGGNs in our research probably led to the statistical insignificance in our result.

The classification of nodule content or uniformity may differ in different studies. According to the consensus statement by the Fleischner Society [27], the ground-glass opacity on CT scans appears as hazy increased opacity of the lung, with preservation of the bronchial and vascular margins. The standard in identifying solid components, which obliterated the bronchial and vascular margins, could be different in different readers [43]. Similarly, variance may exist in the assessment of the nodule content in our work. Therefore, both lung and mediastinal windows were applied in our study for identifying pGGNs and assessing the uniformity of nodule content. In addition, the slice thickness varied in different studies, which may influence the recognition of the nodule components. The difference in methods and materials used in classifying nodule content may affect the result of comparison among the nodules with different pathological diagnoses.

There was no significant difference among the five pathology groups and between IA and NIA with respect to other imaging characteristics: shape, interface, margin, spiculation, vessel relationship, and pleural retraction. This may be partly elucidated by the fact that these features are neither easily found nor strong discriminators in pGGNs < 20 mm.

There are several limitations in this study. First, selection bias could not be avoided in our study. Not all of the patients who underwent surgical excision of GGNs < 2 cm were included. For an optimal evaluation of the morphology of ground-glass nodules, images with a slice thickness of 0.625 mm was one of the inclusion criteria in this retrospective study. The 0.625 mm slice thickness images were obtained on the day of CT-guided localization before surgery. Applying slice thickness of 0.625 mm for evaluating lung nodule in the regular follow-up CT is less practical. Though not all patients who underwent surgery were included in this study, the prevalence of malignancy was 76.2% (138/181) in our study, which is consistent with that of 78% in another report [44]. Second, this is a retrospective cross-sectional study. The longitudinal study with evaluation of nodules’ growth was not performed in this work. Third, the imaging features of pGGNs were interpreted by two radiologists, who reached a consensus without analysis of inter-observer variability. Lastly, this study was based on a single center, and the sample size was rather small. Further study with more cases is required to validate our results.

## 5. Conclusions

The possibility of IA is higher in a pGGN when it is associated with a larger size, lobulation, and air cavity. The air cavity is a significant predictor of IA.

## Figures and Tables

**Table 1:** Demographic data and nodule information.

Variables	Benign	AAH	AIS	MIA	IA	All	*p*-Value
Nodule Numbers	29	14	25	55	58	181	
Age	55.8 ± 10.3	55.4 ± 12.2	55.1 ± 10.9	53.9 ± 11.6	55.4 ± 10.7	55.0 ± 11.0	0.956
Sex (Female)	21 (72.4)	10 (71.4)	16 (64.0)	37 (67.3)	45 (77.6)	129 (71.3)	0.695
Smoking history	2 (6.9)	1 (7.1)	2 (8.0)	8 (14.5)	2 (3.4)	15 (8.3)	0.318
Drinking history	2 (6.9)	1 (7.1)	1 (4.0)	2 (3.6)	2 (3.4)	8 (4.4)	0.833
Cancer history	5 (17.2)	1 (7.1)	3 (12.0)	18 (32.7)	14 (24.1)	41 (22.7)	0.122
Nodule Location							0.698
In upper lobe	15 (51.7)	9 (64.3)	16 (64.0)	27 (49.1)	31 (53.4)	98 (54.1)	
Not in upper lobe	14 (48.3)	5 (35.7)	9 (36.0)	28 (50.9)	27 (46.6)	83 (45.9)	

The numbers in parenthesis indicate percentage. AAH: atypical adenomatous hyperplasia; AIS: adenocarcinoma in situ; MIA: minimally invasive adenocarcinoma; IA: invasive adenocarcinoma.

**Table 2:** Analysis and comparison of computed tomographic findings.

Imaging Features	Benign	AAH	AIS	MIA	IA	*p*-Value
Size-L	7.4 ± 3.0	6.3 ± 2.5	6.0 ± 1.9	7.1 ± 2.7	8.2 ± 3.4	0.029 *
Size-S	5.6 ± 1.9	4.6 ± 1.7	5.0 ± 1.3	5.3 ± 2.5	6.2 ± 2.6	0.043 *
Size-M	6.5 ± 2.4	5.4 ± 2.0	5.5 ± 1.5	6.2 ± 2.5	7.2 ± 2.9	0.025 *
Content						0.643
Homogenous GGN	17 (58.6)	10 (71.4)	16 (64.0)	30 (54.5)	30 (51.7)	
Heterogeneous GGN	12 (41.4)	4 (28.6)	9 (36.0)	25 (45.5)	28 (48.3)	
Shape						0.203
Oval or polygonal	24 (82.8)	12 (85.7)	25 (100.0)	48 (87.3)	53 (91.4)	
Irregular	5 (17.2)	2 (14.3)	0 (0)	7 (12.7)	5 (8.6)	
Interface						0.100
Well-defined	23 (79.3)	13 (92.9)	24 (96.0)	53 (96.4)	51 (87.9)	
Ill-defined	6 (20.7)	1 (7.1)	1 (4.0)	2 (3.6)	7 (12.1)	
Margin						0.708
Smooth	25 (86.2)	12 (85.7)	20 (80.0)	43 (78.2)	43 (74.1)	
Coarse	4 (13.8)	2 (14.3)	5 (20.0)	12 (21.8)	15 (25.9)	
Lobulation	1 (3.4)	1 (7.1)	1 (4.0)	0 (0)	6 (10.3)	0.083
Spiculation	0 (0)	0 (0)	0 (0)	3 (5.5)	1 (1.7)	0.635
Air cavity	8 (27.6)	2 (14.3)	5 (20.0)	11 (20.0)	24 (41.4)	0.060
Vessel relationship						0.532
Type I	26 (89.7)	14 (100.0)	24 (96.0)	47 (85.5)	52 (89.7)	
Type II	3 (10.3)	0 (0)	1 (4.0)	8 (14.5)	6 (10.3)	
Pleural retraction	2 (6.9)	2 (14.3)	4 (16.0)	6 (10.9)	11 (19.0)	0.590

*: *p*-value < 0.05 with statistically significance. The numbers in parenthesis indicate percentage. AAH: atypical adenomatous hyperplasia; AIS: adenocarcinoma in situ; MIA: minimally invasive adenocarcinoma; IA: invasive adenocarcinoma; size-L: the maximal diameter in the long axis; size-S: the maximal short axis diameter perpendicular to the long axis; size-M: the mean value of long and short axis diameters; GGN: ground-glass nodule. Type I vessel relationship was defined as vessels passing by or passing through the nodule; and type II was defined as distorted, dilated, or engorged vessels within the nodule [29].

**Table 3:** Univariate and multivariate analysis between the non-invasive adenocarcinoma (NIA) group and invasive adenocarcinoma (IA) group.

Imaging Features	NIA	IA	Univariate *p*-Value	Multivariate *p*-Value
Size-L	6.8 ± 2.7	8.2 ± 3.4	0.009 *	
Size-S	5.2 ± 2.1	6.2 ± 2.6	0.016 *	
Size-M	6.0 ± 2.3	7.2 ± 2.9	0.008 *	0.051
Heterogeneous content	50 (40.7)	28 (48.3)	0.334	
Irregular shape	14 (11.4)	5 (8.6)	0.572	
Ill-defined interface	10 (8.1)	7 (12.1)	0.397	
Coarse margin	23 (18.7)	15 (25.9)	0.270	
Lobulation	3 (2.4)	6 (10.3)	0.031 *	0.053
Spiculation	3 (2.4)	1 (1.7)	1.000	
Air cavity	26 (21.1)	24 (41.4)	0.004 *	0.042 *
Type II vessel relationship	12 (9.8)	6 (10.3)	0.117	
Pleural retraction	14 (11.4)	11 (19.0)	0.168	

*: *p*-value < 0.05 with statistically significance. The numbers in parenthesis indicate percentage. NIA: non-invasive adenocarcinomas; all benign lesions, atypical adenomatous hyperplasia, adenocarcinoma in situ, and minimally invasive adenocarcinoma are classified to NIA; IA: invasive adenocarcinoma; size-L: the maximal diameter in the long axis; size-S: the maximal short axis diameter perpendicular to the long axis; size-M: the mean value of long and short axis diameters; GGN: ground-glass nodule.

## Data Availability

The data presented in this study are stored in our institutional repository and will be shared on request from the corresponding author.
